# Patient-care time allocation by nurse practitioners and physician assistants in the intensive care unit

**DOI:** 10.1186/cc11195

**Published:** 2012-02-15

**Authors:** David L Carpenter, Sara R Gregg, Daniel S Owens, Timothy G Buchman, Craig M Coopersmith

**Affiliations:** 1 Emory Center for Critical Care, Emory University, 1364 Clifton Road Atlanta, GA 30322, USA; 2 Emory Center for Critical Care and Department of Surgery, Emory University, 1364 Clifton Road Atlanta, GA, 30322, USA

## Abstract

**Introduction:**

Use of nurse practitioners and physician assistants ("affiliates") is increasing significantly in the intensive care unit (ICU). Despite this, few data exist on how affiliates allocate their time in the ICU. The purpose of this study was to understand the allocation of affiliate time into patient-care and non-patient-care activity, further dividing the time devoted to patient care into billable service and equally important but nonbillable care.

**Methods:**

We conducted a quasi experimental study in seven ICUs in an academic hospital and a hybrid academic/community hospital. After a period of self-reporting, a one-time monetary incentive of $2,500 was offered to 39 affiliates in each ICU in which every affiliate documented greater than 75% of their time devoted to patient care over a 6-month period in an effort to understand how affiliates allocated their time throughout a shift. Documentation included billable time (critical care, evaluation and management, procedures) and a new category ("zero charge time"), which facilitated record keeping of other patient-care activities.

**Results:**

At baseline, no ICUs had documentation of 75% patient-care time by all of its affiliates. In the 6 months in which reporting was tied to a group incentive, six of seven ICUs had every affiliate document greater than 75% of their time. Individual time documentation increased from 53% to 84%. Zero-charge time accounted for an average of 21% of each shift. The most common reason was rounding, which accounted for nearly half of all zero-charge time. Sign out, chart review, and teaching were the next most common zero-charge activities. Documentation of time spent on billable activities also increased from 53% of an affiliate's shift to 63%. Time documentation was similar regardless of during which shift an affiliate worked.

**Conclusions:**

Approximately two thirds of an affiliate's shift is spent providing billable services to patients. Greater than 20% of each shift is spent providing equally important but not reimbursable patient care. Understanding how affiliates spend their time and what proportion of time is spent in billable activities can be used to plan the financial impact of staffing ICUs with affiliates.

## Introduction

Optimal patient management in intensive care units (ICUs) engages a multiprofessional team working together to provide consistent, high-reliability care. Frequently a gap exists between actual and optimal care. Only one in three patients in an ICU in the United States is currently treated by a board-certified intensivist [1]. This staffing gap is further heightened by guidelines that specify 24/7 service to ICUs [2,3] and by a limited number of intensivists providing for an aging and increasingly ill population [4,5].

ICUs in academic medical centers commonly rely on resident trainees as caregivers. Because these novices typically serve a 4- to 6-week tour before cycling to their next service, it can be difficult to establish consistent practice and maintain consistent care. The availability of physician trainees in the United States is likely to be further curtailed in the future owing to progressive restrictions on work hours mandated by oversight groups [6,7].

In an attempt to promote more consistent practice with a stable workforce, an increasing number of ICUs have turned to nurse practitioners and physician assistants (collectively referred to hereafter as affiliates) [8-11]. Originally conceived to fill primary care gaps [12,13], affiliates have followed physician specialization with increasing inpatient employment [14]. In 2008, 102,000 nurse practitioners were practicing in the United States [15], of whom 3.9% identified their workplace as inpatient critical care [16]. In 2010 there were 83,466 physician assistants of which 2.2% listed the ICU or critical care as their work place [17]. This suggests that approximately 6,000 affiliates work in ICUs in the United States. These numbers appear to be increasing.

Although the responsibilities of affiliates vary widely between institutions, these duties can include history taking, physical examination, rounding, implementing order sets and protocols, and performing procedures [18-22]. Care provided by affiliates in ICUs and step-down units has been found to be broadly comparable to that of residents [18,22-27]. Although not all affiliates bill for care provided in the ICU, affiliates filed approximately 33,000 Medicare claims in 2008 for the initial hour of adult critical care (CPT 99291) [28].

Despite the increasing use of affiliates in the ICU, few data are available on how their time is allocated. To understand how affiliates spend their time in the ICU, we studied the allocation of affiliate time into patient care and non-patient-care activity, further dividing the time devoted to patient care into billable service and equally important but nonbillable care.

## Materials and methods

### Location

During the study, the Emory Center for Critical Care (ECCC) contained eight ICUs totaling 135 beds contained within a 579-bed academic hospital (Emory University Hospital) and a 511-bed hybrid academic/community hospital (Emory University Hospital Midtown). This includes two medical ICUs, one surgical/transplant ICU, two cardiovascular surgery ICUs, two neuroscience ICUs, and one coronary care unit. The coronary care unit did not employ affiliates until near the end of the intervention and was therefore excluded from this study. Additionally, one of the ICUs was reorganized from a general ICU without dedicated intensivists or affiliates to a neurosciences ICU with dedicated intensivists and affiliates during the preintervention time period. No preintervention data were available for this ICU because of the evolution of its coverage model.

Unless explicitly noted, all ICUs were staffed with affiliates throughout the length of the study. Affiliates were full-time employees of Emory Healthcare, as no independent contractors are employed by the ECCC. Each ICU determined the roles and responsibilities of its own affiliates. The schedule for the affiliates of each ICU was determined by the lead affiliate in each ICU. Before the baseline reporting period, 32 affiliates were employed by the ECCC (including DLC, first author of the article). Throughout the course of the study, another nine affiliates completed orientation, whereas two affiliates left the ECCC. Affiliates who were still in orientation for their ICU (typical range, 3 to 6 months) were not counted in the study. Residents were present in three of the ICUs and contributed to management of approximately 50% of the patients in those ICUs.

### Study design

Baseline billing data were collected from 4/11/2010 to 8/28/2010 (preintervention). Data were analyzed by ICU and further by individual providers. In total, 14,553 patient days and 3,210 patients were counted in the ECCC during this time period.

The intervention was defined as a one-time $2,500 incentive awarded to all affiliates in ICUs that documented 75% of their time devoted to patient care over the 6-month time period from 8/29/2010 to 3/5/2011. The incentive was designed not only to understand how affiliates use their time but also to understand and abide by a process that is intended to maximize efficiency and accuracy of the delivery of quality care. Because high-reliability ICUs require team-based solutions, the incentive was given as a team-based incentive. As such, to qualify for the bonus, all affiliates in a given ICU had to document individually more than 75% patient-care time. This enabled each affiliate to set aside a modest proportion of each shift for non-patient-care related time (conferences, meetings, meals, and so on) while encouraging each affiliate to spend a minimum of 75% of the shift performing duties related to patient care. The decision to use the specific target of 75% time documentation (as opposed to a slightly higher or lower percentage of time) was arbitrary. However, the general rationale behind this target was to ensure that affiliates were, on average, spending most of their shifts performing duties related to patient care, while still allowing them flexibility for academic pursuits and meals. As discussed in detail later, the accounting system promoted the use of "zero-charge" time to account for nonbillable patient-care activities outside of critical care, evaluation and management (E/M), and procedural services rendered by providers to patients. Billable time and zero-charge time counted equally toward the incentive.

Each ICU had a lead affiliate who was provided with weekly updates on both total unit and individual affiliate reporting. Each affiliate also received monthly updates of the ICU and individual performance. During the month-long period between baseline data collection and the intervention, all affiliates received education on time documentation and received weekly updates on their individual patient-care documentation. Care was taken to stress accurate recording of time allocation, including the use of zero-charge time where appropriate. During the 6- month intervention, 20,142 patient days and 4,256 patients were recorded in the ECCC.

### Time documentation

The intervention was designed to encourage accurate documentation of patient care-related time, which included both billable and nonbillable activities. All patient-care time was entered into the computerized billing system IM Bills (Ingenious Med, Atlanta, GA). This system allowed providers to enter time spent providing critical care and E/M services to individual patients as well as time spent performing procedures. As noted earlier, a new category named "zero charge" was created to facilitate accounting of nonbillable patient-care time. Zero-charge time entries brought up a free-text box inviting brief explanation of what activity was performed (examples included time spent rounding, training other providers in procedures, and sign out). Although affiliates were encouraged to enter a reason in the free-text box as to why they were using zero-charge time, this was optional, and some affiliates chose to leave the box blank.

Concurrent and final analyses included the total number of hours of affiliate-provider patient-care time each day, broken down into chargeable (critical care, E/M, procedure) and nonchargeable (zero-charge) time. This was expressed as a percentage of each shift, because affiliates worked shifts ranging from 8 to 13 hours, depending on ICU need. Data on physician patient-care time also collected were throughout the study. Unlike the affiliates, attending intensivists did not work on a shift schedule, so absolute hours per day were collected. No monetary incentive was offered to physicians for time documentation. Of note, because no patient research was performed in this study, this is not characterized as human-subjects research by the Emory IRB, so IRB approval was not necessary to obtain to perform this study.

## Results

### Documentation of patient-care time

During the baseline (preincentive) period, only one of the six ICUs averaged documenting 75% patient-care time (range, 20% to 75%; Table 1). It is important to note that the only way to document time in the baseline period was by billing for patient-care activities. Time that was not billed could have been either (a) patient care-related but not billable (time spent rounding, training other providers in procedures, and sign out), or (b) not patient-care related. No mechanism was in place, however, to determine how much time was dedicated to each of these. The average amount of patient-care time billed was 53% (individual range, 21% to 95%), meaning that at baseline, it was unclear what activities affiliates were engaged in for nearly half of their paid time in the hospital. Even in the ICU that averaged 75% in aggregate, several individual affiliates billed for less than 75% of their time. Only six of 32 affiliates billed for 75% of their time in the preintervention period. Thus before the intervention, no ICU met the goal that each affiliate provider who worked in a specific unit documented spending 75% or greater of their day on patient-care-related activities.

**Table 1:** Baseline time documentation

	Critical care	E/M	Procedure	Patient-care time	Time spent in nonbillable activities
CVICU-1	48%	4%	2%	54%	46%
NICU-1	56%	13%	6%	75%	25%
NICU-2	^a^	^a^	^a^	^a^	^a^
CVICU-2	50%	5%	2%	57%	43%
SICU	20%	16%	3%	39%	61%
MICU-1	9%	1%	10%	20%	80%
MICU-2	38%	4%	1%	43%	57%
**Total**	**40%**	**9%**	**4%**	**53%**	**47%**

^a^Data not available. Nonbillable activities in the baseline period represented a combination of nonbillable patient-care activities and non-patient-care activities. CVICU, cardiovascular ICU; MICU, medical ICU; NICU, neurosciences ICU; SICU, surgical ICU.

**Figure 1:**
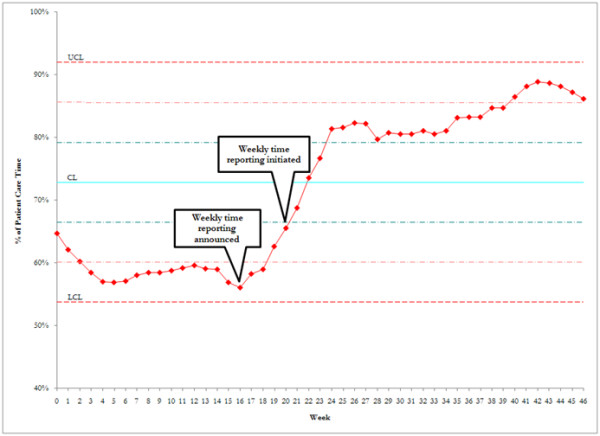
**Effect of intervention on affiliate time reporting**. Reporting was constant before the intervention. After the announcement of the incentive and weekly feedback regarding performance, time documentation increased steadily for the following 6 months.

**Figure 2:**
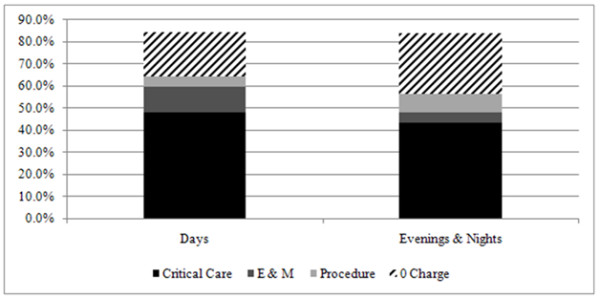
**Effect of shift on time documentation**. Time documentation was similar regardless of which shift an affiliate worked.

After the announcement of the incentive, every affiliate in six of the seven ICUs documented greater than 75% patient-care time, with patient-care time documentation being defined as the combination of billable time and nonbillable but equally important zero-charge time (range, 70% to 89%; Table 2). The average amount of patient-care time documented was 84% (individual range, 61% to 100%), a greater than 30% absolute improvement, with a steady increase in time documentation throughout the 6 months of the intervention (Figure 1). Each individual ICU saw an increase in patient-care documentation, with absolute increases ranging from 12% in the ICU with the highest baseline documentation to 68% in the ICU with the lowest baseline documentation. In total, 34 of 39 affiliates accounted for 75% or greater of their time after the intervention. In total, 25% of affiliates worked either evening or night shift in the postintervention phase. Documentation of patient-care time was similar regardless of which shift an affiliate worked (Figure 2).

**Table 2:** Time documentation after intervention

	Critical care (% change)	E/M (% change)	Procedure (% change)	Zero charge	Portion of patient-care time (% change)	Unaccounted-for time
CVICU-1	49%	8%	2%	28%	87%	13%
	(1%)	(3%)	(0)		(33%)	
NICU-1	62%	10%	7%	8%	87%	13%
	(6%)	(-3%)	(1%)		(12%)	
NICU-2	43%	13%	2%	19%	77%	23%
	^a^	^a^	^a^		^a^	
CVICU-2	48%	8%	2%	27%	85%	15%
	(-2%)	(2%)	(0)		(28%)	
SICU	41%	19%	5%	24%	89%	11%
	(21%)	(3%)	(2%)		(50%)	
MICU-1	28%	2%	18%	40%	88%	12%
	(19%)	(1%)	(8%)		(68%)	
MICU-2	41%	7%	2%	20%	70%	30%
	(3%)	(3%)	(1%)		(27%)	
**Total**	**47%**	**10%**	**6%**	**21%**	**84%**	**16%**
	**(7%)**	**(1%)**	**(2%)**		**(31%)**	

^a^Data not available. Unaccounted-for time after the intervention represented duties not related to patient care, such as meals, conferences, and meetings. CVICU, cardiovascular ICU; MICU, medical ICU; NICU, neurosciences ICU; SICU, surgical ICU.

### Zero-charge time

The key reason to undertake this study was to understand how affiliates spent their time over the course of an average shift. To this end, a new category (zero-charge time) was created to document how nonbillable patient-care time was spent. Zero-charge time entries were accompanied by a text box for the affiliate to provide a free-text explanation of what activity was performed. Overall, zero-charge time accounted for an average of 21% of an affiliate's shift, representing slightly more than 25% of all documented patient-care time. Although 10 different reasons were given for documenting zero-charge time, the most common were rounding with the team while others' patients were being discussed (44%) and sign-out (10%; Table 3).

**Table 3:** Reasons for billing zero-charge time

Comments	Total hours
Rounding	853
No reason stated (free-text box blank)	412
Sign-out	196
Chart review	169
Teaching/training activities	111
Direct patient care	66
Billing time	58
Speaking to family/patient	55
Discharge/transfer-related activities	22
Administrative tasks	9
Patient died	1
**Total**	**1,952**

Convenience sample from December 26, 2010, through January 31, 2011.

### Billable time

Although the intervention was aimed at increasing time reporting as opposed to increasing reimbursement, billable time (defined as the sum of critical care, E/M, and procedures) also increased after the intervention. Because billable patient-care time accounted for all time documentation during the baseline time period, billable time averaged 53% of an affiliate's shift. In the 6 months after the intervention, billable time increased to 63% (individual range, 29% to 92%; Table 4). This represented a 1% to 28% increase per ICU compared with baseline data.

**Table 4:** Billable time

	Preintervention billable time	Postintervention billable time	Pre- vs. postintervention change (%)
CVICU-1	54%	59%	5%
NICU-1	75%	79%	4%
NICU-2	^a^	58%	^a^
CVICU-2	57%	58%	1%
SICU	39%	65%	26%
MICU-1	20%	48%	28%
MICU-2	43%	50%	7%
**Total**	**53%**	**63%**	**10%**

^a^Data not available. CVICU, cardiovascular ICU; MICU, medical ICU; NICU, neurosciences ICU; SICU, surgical ICU.

Billable time was 8% higher for day-shift affiliates compared with evening/night-shift affiliates (Figure 2). Whereas critical care billing was generally similar between the groups, day-shift affiliates billed more E/M time (11% versus 5%) and less procedure time (5% versus 9%). Physician time documentation

**Figure 3:**
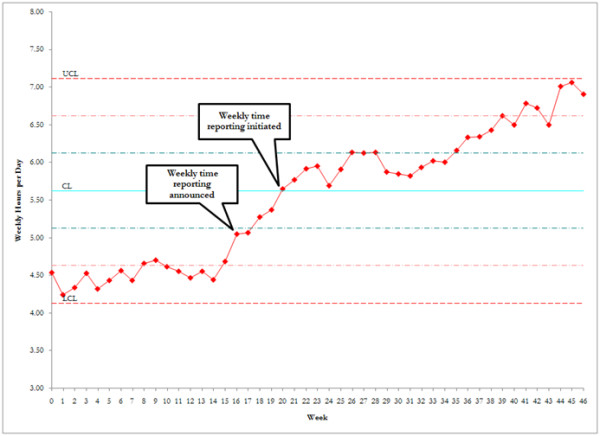
**Effect of affiliate intervention on physician time reporting**. Reporting was relatively constant before the intervention. After the announcement of the affiliate incentive, physician time documentation increased steadily for the following 6 months, although no incentive was offered to the attending staff.

Physician billing time was also collected simultaneous to affiliate time documentation. Before the intervention, physician billable time averaged 4.7 hours per day (individual range, 2.7 to 10.8 hours). Although no monetary incentive was offered to intensivists, physician billing time increased in parallel to affiliate time documentation, increasing to 6.3 hours per day after the intervention (individual range, 2.7 to 10.7 hours; Figure 3).

## Discussion

With the shortage of intensivists and recognition of the need to provide consistent care, many ICUs are increasingly turning to affiliates [18,29]. Although previous studies have examined affiliate time on a surgical service [30], it has been unclear how affiliates apportion their time during the course of an ICU shift. This study demonstrates that, on average, affiliates spend nearly 85% of their time on patient-care-related activities. Of this, three fourths of the time corresponds to billable services, whereas one fourth is not billable.

This information is useful on a number of levels. When a hospital is considering implementing an affiliate program in the ICU, it offers an estimate for financial modeling. The economic impact of affiliates in outpatient medicine has been studied [31,32], but little is known about the impact in the ICU. This analysis anticipates how much reimbursement should be modeled for each provider. Regardless of the time of day, a typical affiliate bills for about two thirds of the time present in the hospital. Nonbillable time, although important to patient care, must be accounted for in the budgeting process.

We believe this study represents the first description of how affiliates practicing in ICUs allocate their time, with a special focus on the important but nonbillable services that they render. Approximately half of this time is spent rounding. Attendings and affiliates cannot independently bill for time spent rounding on patients together, unless they are performing different tasks. Whereas communication and decision making make it important for physicians and affiliates to be together on rounds, it is equally important when building an ICU budget to account for this time. After rounding, the most common nonbillable patient-care tasks were sign out, chart review, and teaching/training activities (Table 3). Having effective provider-to-provider sign-out is critical to patient safety. Because both providers cannot simultaneously bill for this sign-out, this represents a necessary yet nonreimbursable cost required to provide quality patient care. Teaching represents a potential underappreciated part of the affiliate's shift. Whether this teaching involves training new affiliates or resident physicians, teaching is part of the core mission of an academic medical center and must be encouraged. We note that "chart review" sometimes did not result in billable time. This apparent paradox seemed related to follow-up care (such as review of consultant notes or laboratory or image data) of non-critically ill patients who had E/M time billed by another provider earlier in the calendar day.

The results also allow a comparison of how different ICUs use their affiliates. For example, the ratio of time spent on critical care billing to E/M billing as well as the actual values was fairly similar in six of the seven ICUs. However, in one ICU (MICU-1), affiliates documented nearly twice as much zero-charge time while accounting for considerably less critical care and E/M time. This suggests that the tasks of affiliates in this single ICU may have been different from that seen in the other six ICUs. Additionally, the single ICU that did not qualify for the monetary incentive (MICU-2) had a similar amount of zero-charge time to that of the other ICUs but lagged behind in billable time. This suggests that affiliates were being tasked differently or were incompletely documenting patient care that they rendered.

An unexpected outcome of the study was the increase in physician in time documentation. During the baseline period, physician billing, like affiliate time documentation, was relatively flat (Figure 3). Before the intervention, we hypothesized that rigorous records of affiliate time might lead to a reciprocal decrease in attending time. However, after the intervention, attending time documentation increased in parallel (compare Figure 1 with Figure 3) despite the lack of incentive for the attending staff. We speculate that this "halo" effect was the result of intense education and regular feedback to all providers, attending and affiliate, during the intervention. It should be noted, however, that the range of individual physician time-accounting practices did not change throughout the entire study (range, 2.7 to 10.7 hours per day both before and after the intervention), suggesting that some physicians can further improve their time accounting.

The strategy used in this study to obtain improved documentation of patient-care time was a group incentive to all affiliates in an ICU, which was earned only if all members of the group improved their performance to reach the threshold. We again emphasize that the incentive encouraged the use of zero-charge documentation to help the community understand the many valid uses of provider time that are nevertheless not appropriate for a charge. The sole expectation was accuracy in recording time allocation.

The use of incentives as a method to improve process outcomes is increasingly prevalent in medicine [33,34], although results have been mixed, with a variety of unintended consequences [35-39]. In contrast to this study, most incentive programs are aimed at individual providers. In the fields of compensation and economics, a number of studies have examined the interaction of group and individual incentives. Relatively little examination of group incentives in healthcare has been undertaken. However, high-reliability care in critical illness requires optimal function of all members of the ICU team, and therefore, a clear intent of this study was to motivate the affiliates to work toward a common good.

This study has a number of limitations. The study was conducted during a time of significant expansion within the ECCC, as new affiliates were actively being hired during the baseline period and the intervention period. Orienting these new employees may have changed the daily experience of affiliates from what it will be once a stable team is in place in each ICU. Although orientation was a relatively small component of zero-charge time, anecdotally, many affiliates reported a significant impact in how they distributed their time daily. Because Table 3 represents a convenience sampling of zero-charge time, this may not have been reflective of zero-charge time for the remainder of the study.

Another limitation lies in the fact that the study relied on self-reported allocations of time. This is because we chose not to use trained observers or a punch clock because we wanted to minimize disturbance to ordinary workflows. However, sampling of presence and activity in the ICU strongly suggested correspondence between observed activity and reporting. The accuracy of self-reporting was also supported by the consistency of zero-charge reporting between the majority of the ICUs (Table 2) and the fact that the specific breakdown of zero-charge time was similar between the ICUs (data not shown). Having a formal mechanism to improve documentation, such as providing each affiliate and physician with a handheld computer, is an area for process improvement in the future.

A discrepancy was found between how affiliate time was calculated (percentage of shift) and physician hours were calculated (hours per day). The rationale behind this was that affiliates had fixed shifts, whereas attending physicians did not. However, because many affiliates worked past the end of the salaried shift to complete patient care, it was possible to document more time than the provider was scheduled to work, which could theoretically overestimate the percentage of each shift dedicated to patient care, although it would not alter the actual number of hours devoted to patient care.

A simultaneous strength and weakness of the study was the relative lack of homogeneity among the ICUs. In addition to having different patient populations, the ICUs had a wide variety of coverage models, dependent in part on availability of residents and in part on how the ICU deployed its affiliates. For instance, in some ICUs, a separate affiliate service and resident service existed. In other ICUs, only a single service consisted of attendings and affiliates or attendings, affiliates, and fellows/residents. Additionally, even in ICUs with separate affiliate and resident services during the day, patient-management decisions could be shared or distinct at night, depending on the acuity, coverage model, and staffing of the ICU. This led to a range in which affiliates could manage or co-manage between six and 27 patients, either on their own service or as part of a larger team, depending on individual ICU and shift. Although this led to a range of behavior, it also likely increased the "real world" significance of the findings.

Finally, patient outcomes were not examined as part of this study, so no conclusion can be drawn regarding the quality of care provided by the affiliates in the study. Further studies are needed to demonstrate whether these findings are generalizable to other medical centers.

## Conclusions

Approximately two thirds of an affiliate's shift is spent providing billable services to patients, whereas greater than 20% of each shift is spent providing equally important but nonreimbursable patient care. The most common nonreimbursable services performed are rounding, sign-out, chart review, and teaching. Although all patient-care time is important to patient care, understanding the relation between billable and nonbillable time should be useful for financial planning for hospitals interested in incorporating affiliates into their coverage model.

## Key messages

• Greater than 20% of an affiliate's shift is spent delivering important, but nonreimbursable, patient care

• The most common nonreimbursable activities performed by affiliates are rounding, sign out, chart review, and teaching

• Two thirds of an affiliate's shift is spent in delivery of billable (critical care, E/M, procedure) services

• Understanding how affiliates spend their time and what proportion of time is spent in billable activities can be used to plan the financial impact of staffing ICUs with affiliates.

## Abbreviations

CVICU: cardiovascular ICU; ECCC: Emory Center for Critical Care; E/M: evaluation and management; ICU: intensive care unit; MICU: medical ICU; NICU: neurosciences ICU; SICU: surgical ICU.

## Competing interests

The authors declare that they have no competing interests.

## Authors' contributions

DC analyzed and interpreted the data and drafted the manuscript. SG analyzed and interpreted the data and critically revised the manuscript for important intellectual content. DO was responsible for study concept and design, analyzed and interpreted the data, and critically revised the manuscript for important intellectual content. TB was responsible for study concept and design, analyzed and interpreted the data, critically revised the manuscript for important intellectual content, and supervised the overall study. CC was responsible for study concept and design, analyzed and interpreted the data, drafted the manuscript, and supervised the overall study. All authors read and approved the manuscript for publication.
